# The Influence of the Modification of Carbon Nanotubes on the Properties of Copper Matrix Sintered Materials

**DOI:** 10.3390/ma17061427

**Published:** 2024-03-20

**Authors:** Adam Piasecki, Julia Sobkowiak, Dariusz Boroński, Katarzyna Siwińska-Ciesielczyk, Piotr Paczos

**Affiliations:** 1 Institute of Materials Science and Engineering, Faculty of Materials Engineering and Technical Physics, Poznan University of Technology, Jana Pawla II 24, 61-139 Poznan, Poland; juliasobkowiak5@gmail.com; 2 Faculty of Mechanical Engineering, Bydgoszcz University of Science and Technology, 85-796 Bydgoszcz, Poland; dariusz.boronski@pbs.edu.pl; 3 Institute of Chemical Technology and Engineering, Faculty of Chemical Technology, Poznan University of Technology, Berdychowo 4, 60-965 Poznan, Poland; katarzyna.siwinska-ciesielczyk@put.poznan.pl; 4 Institute of Applied Mechanics, Faculty of Mechanical Engineering, Poznan University of Technology, Piotrowo 3, 61-138 Poznan, Poland; piotr.paczos@put.poznan.pl

**Keywords:** sinters, carbon nanotubes, wear resistance, tribology, COF, mechanical properties

## Abstract

This paper presents the results of research on the microstructure, mechanical, and tribological properties of Cu/0.5 wt.% MWCNT (multi-walled carbon nanotube) sintered composite materials produced by powder metallurgy. The purpose of this research was to investigate the impact of carbon nanotube modifications on the uniformity of their dispersion and the effectiveness of their bonding with the matrix. The MWCNTs were modified by chemical oxidation. Additionally, a modification of the ingredient mixing method utilizing ultrasonic frequencies was employed. The tests were carried out using scanning electron microscopy (SEM), transmission electron microscopy (TEM), Vickers hardness tests, static compression tests, and wear tests using the pin-on-disc method. Furthermore, mechanical properties and strain distribution analyses of the micro-specimens were conducted using the Micro-Fatigue System (MFS). The implemented modifications had a positive effect on the dispersion of MWCNTs in the copper matrix and on the mechanical and tribological properties of the sinters.

## 1. Introduction

Manufacturing self-lubricating, wear-resistant composite materials containing solid lubricants enables the resolution of numerous issues associated with commonly used lubricating oils and greases on a mass scale. The environmental contamination associated with their production and usage is eliminated. There is no need for frequent replenishment of the lubricant, which opens up new possibilities for applications in advanced industrial and scientific fields. The applications include their use at high temperatures [1,2]. The use of carbon nanotubes (CNTs) as a solid lubricant has potential to enhance the mechanical properties of composites while simultaneously reducing their mass. It represents one of the most efficient and cost-effective methods for increasing the durability of machine components [3,4,5].

Copper, as a matrix, provides high thermal conductivity and facilitates the efficient dissipation of heat from the friction area of the mating parts. Merging copper and CNTs promises a composite with superior performance due to the CNTs’ unique nanoscale properties [6,7,8,9]. Despite over three decades of research on their synthesis and processing, their potential is still not being fully utilized, which is crucial for the promise to be accomplished.

Carbon nanotubes are built of coiled single or multiple coaxially arranged graphene sheets. The cylindrical structure of coiled graphene imparts its novel properties. Compared to graphene, carbon nanotubes exhibit greater thermal and electrical conductivity (nearly non-resistive) and better mechanical properties [10,11,12,13,14,15], with an elastic modulus of approx. 1 TPa and a tensile strength of 150 GPa. Furthermore, their surface exhibits enhanced chemical and biological activity [16]. In self-lubricating metal matrix composites, nanotubes contribute to enhanced performance, exceeding the properties of graphene. Their unique structure influences the improvement in bearing capabilities by integrating with the tribopair in two ways, depending on the contact pressure force. At low contact pressure, nanotubes maintain their cylindrical shape while rolling between the tribopairs. Under high contact pressure, they undergo deformation, forming a protective film that reduces the coefficient of friction [17]. The lubrication efficiency depends on the diameter and length of the nanotubes. Their proper dispersion and, consequently, uniform release from the matrix must be ensured to achieve the best self-lubricating properties of the composite.

In the process of manufacturing Cu/CNT composites, two main issues arising from the nature of both components need to be addressed. For pure copper to have better properties, the composite must exhibit appropriate interfacial bonding between the constituents, which poses a significant challenge because of the nearly zero wettability and solubility of carbon nanotubes in copper. This issue is being tackled through the modifications, enabling the formation of an interfacial transition zone between the reinforcement and the matrix [18,19,20]. The tendency of nanotubes to form agglomerates poses a challenge in obtaining a composite with the appropriate dispersion of the components [21]. Powder metallurgy and nanotube modifications allow the control of their distribution within the matrix and interaction with copper. In the works [7,22], carbon nanotubes were modified by decorating them with copper. MWCNTs were coated with copper nanoparticles to increase their weight and prevent them from escaping to the surface during the preparation of powder mixtures and to increase their adhesion to metallic grains. Theoretical properties of a single nanotube cannot be directly compared with those of other materials (copper) on a macroscopic scale. The practical properties of nanotubes are largely dependent on their arrangement and dispersion. The majority of currently known modification methods are costly and inefficient, which constitutes a barrier hindering the transfer of these technologies on an industrial scale. Therefore, further research is crucial in identifying directions leading to the development of materials approaching the characteristics of ideal Cu/CNTs composites.

In this work, a Cu/CNT composite was produced by powder metallurgy. The multi-walled carbon nanotubes were modified by chemical oxidation. MWCNTs were functionalized to improve the strength of their bonding to the metal matrix through the formation of functional groups –COOH and –OH on the surface of nanotubes [23]. The modified powder was combined with a copper matrix in an ultrasonic chamber.

## 2. Materials and Methods

### 2.1. Chemical Oxidation of MWCNTs

**Figure 1:**
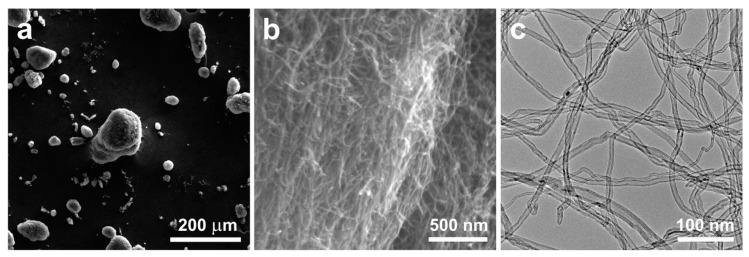
Agglomerates of raw multi-walled carbon nanotubes: SEM (**a**,**b**), TEM (**c**).

This research was conducted using multi-walled carbon nanotubes NC7000™ (NANOCYL^®^, Sambreville, Belgium), produced through catalytic chemical vapor deposition with an average diameter of approximately 11 nm (Figure 1).

Some of the carbon nanotubes were chemically oxidized using concentrated sulfuric (95%, Chempur) and nitric acid (65%, AnalaR Normapur). In the first stage, a mixture of sulfuric (VI) and nitric (V) acid was prepared in a volume ratio of 3:1, into which the carbon nanotubes were introduced. The chemical oxidation process was carried out in a water bath at 50 °C for 3.5 h. The reaction system was stirred intensely throughout the reaction using a Eurostar Digital high-speed mixer from Ika Werke at a speed of 350 rpm. After the reaction, the reaction system was transferred to a beaker containing 3000 cm^3^ of deionized water and left for 20 h. In the next step, filtration and washing processes were carried out until neutral pH was obtained. The final unit operation was to dry the resulting filter cake at 50 °C for 12 h.

### 2.2. Preparation of the Powder Mixtures and Ultrasonication

To prepare the mixture, copper powder AMIL (Werkstofftechnologie GmbH, Chemnitz, Germany) with a purity of 99.7%, was used. The copper powder was produced using the electrolytic method, exhibiting dendritic morphology, with an average grain size of 75 μm. The carbon nanotubes were added into a crystallizer containing isopropyl alcohol (p.a.) and placed in the POLSONIC ultrasonic chamber. The nanotube suspension was then subjected to 40 kHz ultrasonic treatment at room temperature for 5 min. Subsequently, the copper powder was added to the crystallizer and ultrasonic mixing was continued for another 15 min. Agglomerates of modified carbon nanotubes can be separated before or during mixing with the copper matrix using ultrasound [24]. Ultrasonic waves produce rapidly collapsing cavitation bubbles, causing separation and shear forces. Untangled nanotubes cut into shorter lengths mix more easily with the copper powder and anchor around its grains. The resulting mixture was left at room temperature for complete alcohol evaporation. The powder was then mixed in a ceramic mortar grinder. The powder mixture contained 0.5 wt.% multi-walled carbon nanotubes.

### 2.3. Consolidation and Sintering

The powders were uniaxially compacted using the hydraulic press IR-PRESS-25T MP250 (MAASSEN GmbH, Möglingen, Germany) in a die with a 4 mm diameter under at a pressure of 1.17 GPa. The loading time was 30 s. The compacts were heated at a rate of 300 °C/h and subsequently sintered at 750 °C for 3 h in a tube furnace (R50/250/13, Nabertherm GmbH, Lilienthal, Germany) under an argon atmosphere. The sinters were cooled down inside the furnace. The temperature of 750 °C corresponds to approximately 0.7 times the melting temperature of the main component of the sintered material. The pure copper sinters were marked with A. The produced sinters contained unmodified (B) or modified (C) multi-walled carbon nanotubes.

### 2.4. Powder Morphology Characteristics

Observations of the morphology of the MWCNT powders and Cu/MWCNT mixtures after modification were carried out using the MIRA 3 (Tescan, Brno, The Czech Republic) scanning electron microscope with secondary electron contrast. The accelerating voltage of 12 kV was applied. The Titan G2 60-300 kV (FEI, Lausanne, Switzerland) transmission electron microscope was also employed. Microscopic examinations were conducted at an accelerating voltage of 300 kV. The TEM imaging of the MWCNT microstructure was performed in the bright-field mode using a CCD camera as the detector.

### 2.5. Mechanical and Tribological Testing Methods

The micro-specimen tests were performed using the MFS (Micro-Fatigue System) dedicated to static and fatigue tests of micro-objects [25,26]. The load on the specimens in the MFS system is applied using a nano- or a micro-drive. In the first case, the specimen is loaded with a piezoelectric actuator with a displacement resolution of 1.7 nm. The second drive system with a resolution of 1 μm is based on a precise electromechanical drive.

**Figure 2:**
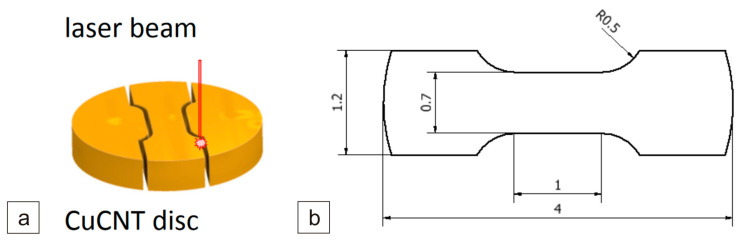
Schematic procedure for extracting specimens from the MWCNT/Cu sinters (**a**), dimensions of the tensile test specimen (**b**).

Micro-specimens for MFS were prepared by employing a laser micro-machining technique. This method involved subjecting the specimen outline to multiple iterations (ranging from several to tens of thousands) of low-power laser beam passes (Figure 2a). This approach served to mitigate potential temperature-induced alterations in the microstructure of the specimens.

The mechanical properties of the MWCNT/Cu composites were tested using the micro-specimens taken from prepared disks with a diameter of 4 mm and an average thickness of 0.7 mm. For each type of composite tested, three specimens were made with the shapes and nominal dimensions shown in Figure 2b.

Due to the very small dimensions of the measurement area of the specimens, averaging approximately 1.0 × 0.7 mm, the digital image correlation (DIC) method was employed for strain measurement in the MFS system [27]. This method involves strain measurement based on the natural image of the specimen surface without the application of additional markers. The surface image of the specimen is observed using high-quality telecentric lenses (VS Technology, Tokyo, Japan) with micrometric optical resolution and a high-resolution Basler Ace camera. The DIC method described above was also used to analyze the full-field strain distribution in the specimen during the tests.

The sinters were subjected to a uniaxial static compression test using the Z100 machine (ZwickRoell, Ulm, Germany). The aim of the test was to determine the force–deformation characteristics, Young’s modulus, and strain energy values. The hardness of the sinters was measured using the Vickers method with a 5 kG load and a dwell time of 15 s on the FALCON 500 (INNOVATEST, Maastricht, The Netherlands) automatic hardness tester. The 5 kG load was selected in order for the indentation to encompass both phases of the composite, thus yielding a more representative hardness measurement.

The measurement of the tribological properties of the sinters at room temperature was carried out on a tribometer (T-21, Lukasiewicz Research Network—IST, Radom, Poland) in a pin-on-disc arrangement. The frictional tests under dry friction conditions were carried out under a load of 0.5 kG at room temperature, a rotational speed of 140 rpm, and a wear track diameter of 7 mm. The counter-specimens in the form of discs had a diameter of 25.4 mm and a height of 4 mm. The pins had a diameter of 4 mm and a height of 5 mm. The counter-specimen was an Inconel^®^625 alloy disk that was initially ground with 80-grit paper to facilitate the observation of the wear tracks against a homogeneous background of unidirectional scratches. The observations of the friction tracks before and after the removal of the wear products were carried out using a MIRA3 (Tescan, Brno, The Czech Republic) scanning electron microscope. Additionally, chemical composition tests were performed using the EDS X-ray microanalyzer (Ultim Max 65, Oxford Instruments, Abingdon, UK).

All of the tests described above were repeated three times.

## 3. Results

**Figure 3:**
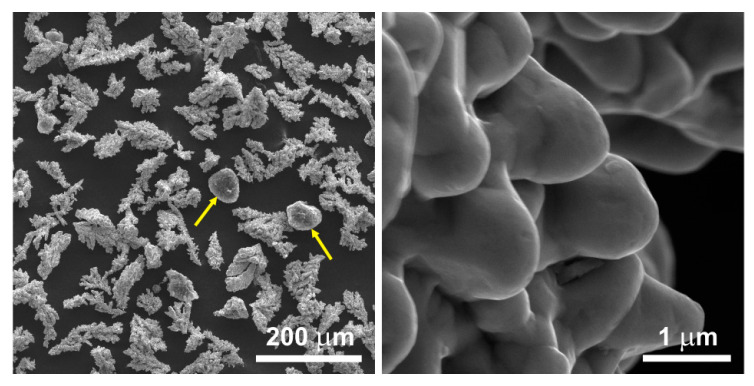
The structure of the 0.5 wt.% MWCNT/Cu powder after mixing in a ceramic mortar grinder. The arrows indicate the agglomerates of the nanotubes.

The raw MWCNT powder existed solely in the form of spherical agglomerates of intertwined nanotubes attracted to each other by the van der Waals forces. Upon mixing with the copper powder, the nanotubes did not disperse and did not effectively bond with the matrix. Consequently, following the mixing of the raw MWCNT powder with the copper powder in a ceramic mortar grinder (Figure 3), no significant dispersion of nanotubes amidst the copper powder grains was observed. The spherical agglomerates of unmodified MWCNTs remained intact during the mixing process, thus failing to utilize the properties arising from the unique shape of the nanotubes.

**Figure 4:**
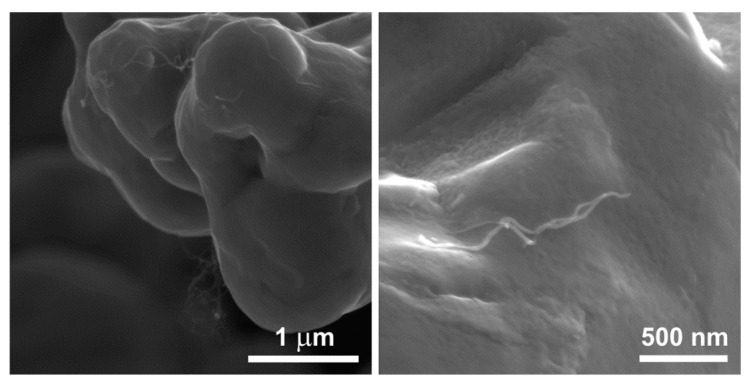
The structure of the 0.5 wt.% MWCNT/Cu powder mixed in an ultrasonic chamber and a ceramic mortar grinder.

The ultrasonic chamber-assisted mixing of the powder containing unmodified MWCNTs resulted in the production of a powder with distinctly separated nanotubes entwining the copper dendrites (Figure 4). This enhanced the dispersion of the nanotubes within the copper powder. The quantity of the separated fibers, however, remained relatively limited.

**Figure 5:**
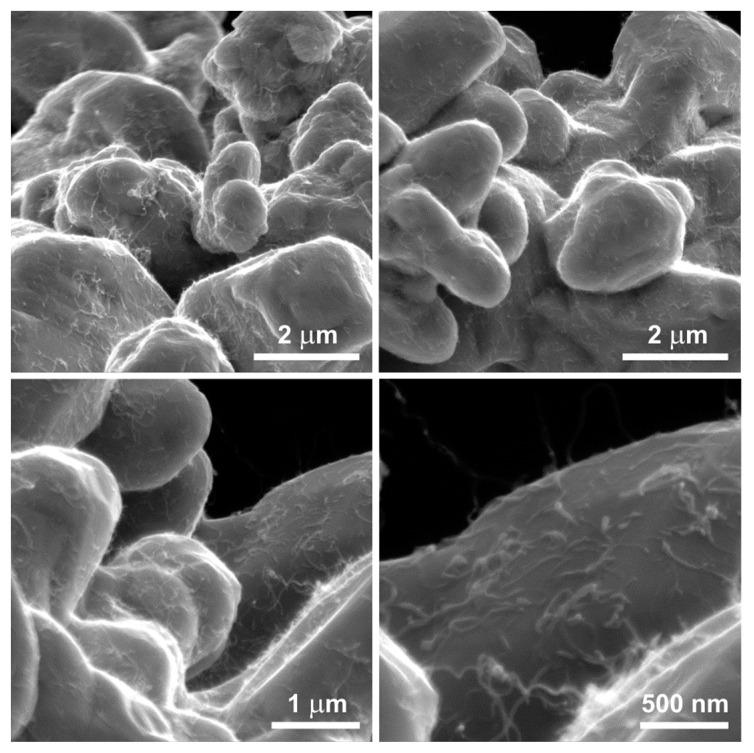
The structure of the 0.5 wt.% MWCNT/Cu powder containing chemically modified nanotubes, mixed in an ultrasonic chamber and a ceramic mortar grinder.

Figure 5 shows the effect of untangling the agglomerates through the described chemical modification and ultrasonic chamber-assisted mixing method. A greater number of individual nanotubes can be observed on the copper dendrites compared to Figure 4. In [28], the authors have shown the dispersion of the nanotubes in the unmodified 0.5 wt.% MWCNT/Cu powder processed by ultrasonication and high-energy attritor milling, which is visibly worse than the dispersion of the acid-treated MWCNTs used in the present study.

The separation of the agglomerates led to the production of a powder with distinctly separated MWCNT fibers evenly covering the entire surface of the copper dendrites. Individual fibers adhere more effectively to the grains, forming a uniform mesh similar to a spider’s web. The favorable coverage of the copper dendrites by the disentangled carbon nanotubes can be substantiated by the formation of oxygen-containing functional groups that cause an increase in the hydrophilicity on the surface of the carbon nanotubes [29]. However, prolonged acidification of MWCNTs may have a destructive effect on their tubular structure [19].

**Figure 6:**
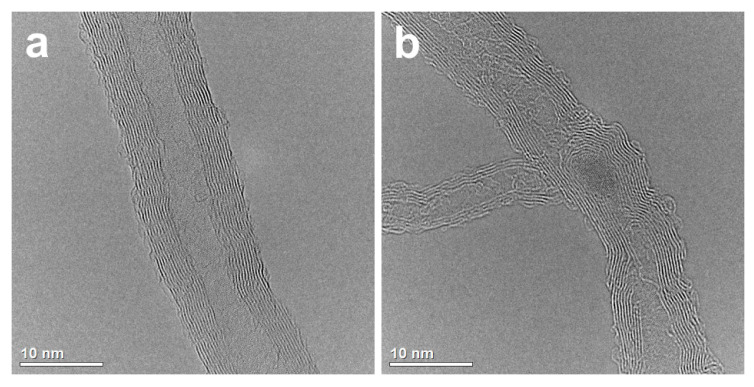
TEM image of the unmodified MWCNT (**a**) and the chemically oxidized one (**b**).

In the present study, the observation using transmission electron microscopy revealed differences in the surface structure of carbon nanotubes before and after modification. The surface of the unmodified nanotubes is relatively smooth and lacks many defects. Modifying the nanotubes through chemical oxidation leads to the formation of defects and functional groups containing oxygen on their surface. Based on Figure 5 and Figure 6b, it can be inferred that the formation of bonds between copper and the functional groups occurred, resulting in the most favorable bonding of the powder components.

A sintered composite produced from a mixture of the unmodified MWCNT powder and the copper powder blended in an ultrasonic chamber and a ceramic mortar grinder reached an average hardness of 31.31 HV5. This outcome represents a 19% increment compared to the hardness of a pure copper sinter (Table 1). Nonetheless, the standard deviation observed across all measurements signifies a lack of uniformity within the sintered structure. Thus, the mere utilization of the ultrasonic chamber for mixing non-modified MWCNTs and the copper powder does not yield adequate dispersion of the reinforcing phase within the matrix.

A sintered Cu/MWCNT composite containing modified nanotubes has a similar average hardness of 31.30 HV5 (Table 1). The standard deviation is relatively low, indicating a homogeneous distribution of the reinforcing phase within the sintered materials. The chemical modification of the powder has led to favorable blending of the sinter constituents.

**Figure 7:**
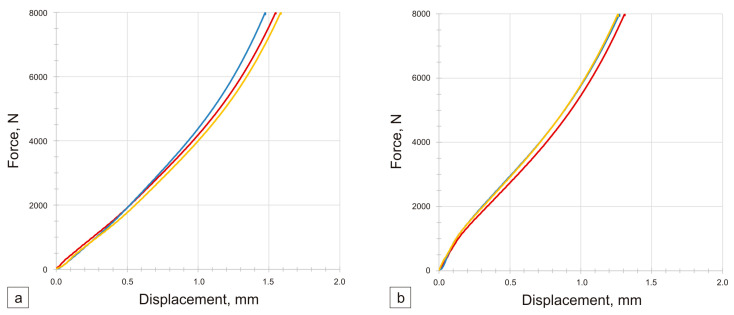
Force–deformation characteristics of 0.5 wt.% MWCNT/Cu sinter containing unmodified (**a**) and chemically modified nanotubes (**b**) mixed in an ultrasonic chamber and a ceramic mortar. Compression test.

The graphs obtained following the static compression test exhibit the characteristic shape of a plastic material (Figure 7). The cylindrical sinters underwent significant deformation, changing their shape to barrel-like. The mechanical properties of nanotubes enable the fabrication of a sinter that yields more advantageous results than a sinter containing 100% pure copper. A significant initial rigidity of the sintered material can be observed. The results of three measurements are not divergent, thus indicating the obtainment of three specimens with homogeneous properties. Additionally, the average relative change in the solid volume during compression is relatively minor (approximately 13%), thus signifying the absence of numerous voids in the structure induced by unbroken agglomerates of the nanotubes. Based on the compression test, no significant differences were observed in the stiffness of the sinters containing modified MWCNTs compared to pure copper ones. In the case of sinters containing unmodified MWCNTs, the stiffness is lower by approximately 5% compared to sinters containing pure copper and sinters containing modified MWCNTs. It was found that the work of the axial deformation at 8 kN (max. test load) was higher for sinters containing unmodified and modified MWCNTs by 29% and 21%, respectively, compared to pure copper sinters.

**Figure 8:**
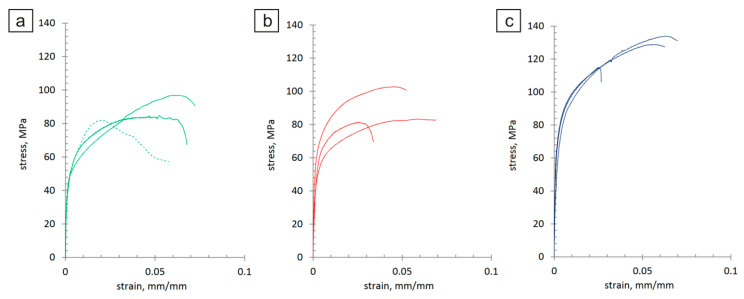
Stress–strain graphs for pure copper sinters (**a**), sinters containing unmodified MWCNTs (**b**), sinter containing modified MWCNTs (**c**). Tensile test.

The static tensile test of the micro-specimens was conducted at a constant displacement rate of 0.0009 mm/s. The measurement of elongation was performed based on a 1 mm gauge length. Additionally, strain distributions throughout the measurement area of the specimens were determined independently of the elongation measurement. The stress–strain graphs recorded during the tests for individual specimen types are presented in Figure 8. Based on the tensile test, it can be observed that the curves overlap for the sinters containing modified carbon nanotubes. This proves the even distribution of the carbon nanotubes in the copper matrix. In the case of pure copper sinters and sinters containing unmodified nanotubes, the curves are different.

**Figure 9:**
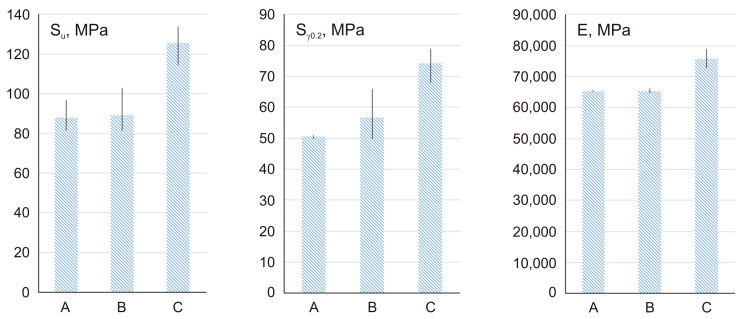
Graphic comparison of the mechanical properties of the sinters, Cu (A), 0.5 wt.% unmodified MWCNTs/Cu (B), 0.5 wt.% modified MWCNTs/Cu (C).

Based on the recorded graphs, the values of the yield strength S_y0_._2_ and immediate tensile strength Su were determined (Table 1, Figure 9). This table also provides the values of the longitudinal elastic modulus E (Young’s modulus) calculated for the linear portions of the stress–strain characteristics. Sinters containing modified nanotubes are characterized by the highest values of yield strength, tensile strength, and Young’s modulus. An over 40% increase was observed in the average immediate tensile strength of the modified specimens compared to the average strength of the 100% Cu reference ones. Similarly, the comparison of the yield strength shows an increase of over 45% and Young’s modulus values of approximately 15%.

**Figure 10:**
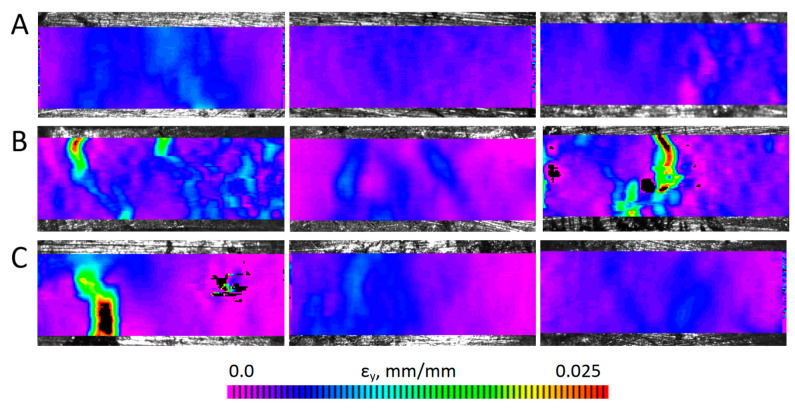
Strain distributions ε_y_ in the specimens, determined for the stress level corresponding to the nominal yield strength. Cu (**A**), 0.5 wt.% unmodified MWCNTs/Cu (**B**), 0.5 wt.% modified MWCNTs/Cu (**C**).

Images of the specimens recorded during the static tensile tests were analyzed with the digital image correlation method, which enabled the determination of the strain distributions occurring in the specimens for the increasing load values. Example strain distributions ε_y_ in the loading direction determined for individual specimens at nominal stress corresponding to the yield strength are shown in Figure 10. For easier comparison, the same color scale was applied to all specimens. For the sinter containing unmodified carbon nanotubes, strong local strain accumulations could be observed, which indicates the possibility of local cracks/delaminations of the specimen surfaces significantly preceding the decohesion phase of the specimen in its entire volume. In the case of the sinter containing modified carbon nanotubes, the strain concentrations are minimal, similar to those of the pure copper sinter. The exception is the first type C specimen, in which the local strain concentration is associated with the emerging crack and a lower ultimate tensile strength compared to other specimens of this type, which can be seen in Figure 8c. However, also in this case, the strain distributions determined in the earlier phases of loading were more uniform in nature compared to type B specimens.

**Figure 11:**
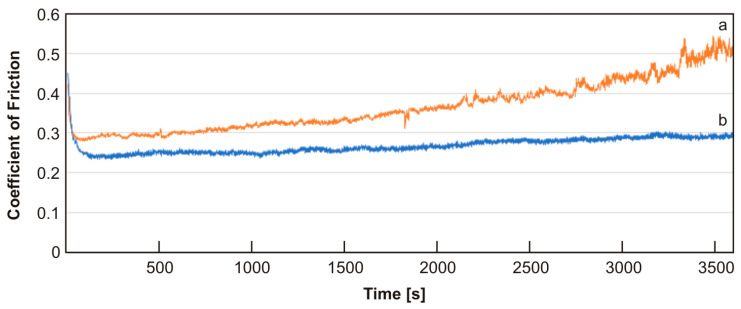
Coefficient of friction vs. time of the friction pair Inconel^®^625–Cu + unmodified 0.5 wt.% MWCNT sinter (**a**) and Inconel^®^625–Cu + modified 0.5 wt.% MWCNT sinter (**b**).

The results of the wear tests are presented in the form of graphs showing the friction coefficients vs. the time of friction (Figure 11).

For the Inconel^®^625–Cu + unmodified 0.5 wt.% MWCNT friction pair, an increase in the coefficient of friction was observed during the test from approx. 0.3 to 0.5. Furthermore, significant fluctuations in the value of the coefficient of friction were observed after approximately 2100 s, which intensified particularly after 3100 s. This is probably related to the appearance of adhesive wear.

For the friction pair cooperating with the sinter containing 0.5 wt.% modified MWCNTs, small fluctuations in the value of the coefficient of friction were observed during the wear test. The coefficient of friction slightly increased over the course of the test, stabilizing at approximately 0.27. The use of sinters containing modified carbon nanotubes in the copper matrix causes a 4-fold reduction in the value of the coefficient of friction compared to pure copper sinters, for which the COF in the friction pair with the Inconel^®^625 alloy was 1.08 [7].

**Figure 12:**
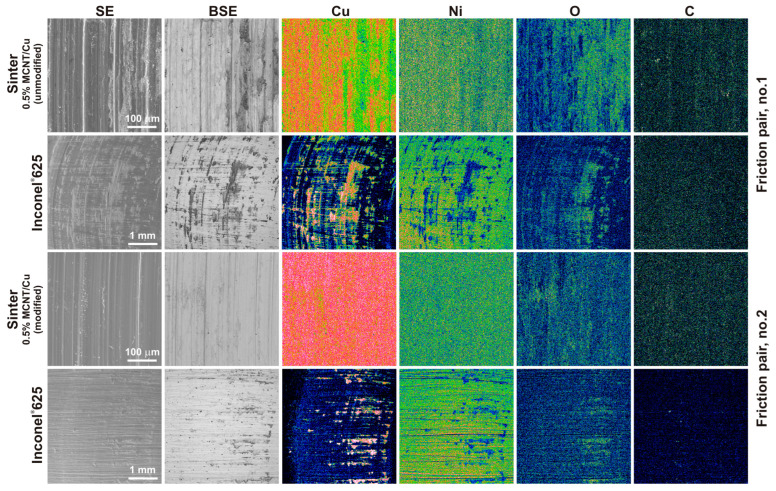
Worn surfaces of the sinters and the counter-specimens (EDS maps).

Figure 12 presents the results of the SEM observations and chemical composition tests in the form of EDS maps of the worn surface of the friction pairs. The SEM images were taken in the SE contrast to observe the changes on the surface of the sinters and counter-specimens. The BSE contrast was intended to observe differences in the chemical composition, confirming the adhesive or oxidative wear. EDS maps were used to determine the locations of Cu, Ni, O, and C on these surfaces. Based on the observations made on the worn surface of the sintered materials, it was found that the main mechanism of their wear was wear caused by microcutting, with the microgrooves on the worn surface of the sinter containing modified MWCNTs being less visible. The plastic deformations on the worn surfaces suggest wear caused by micro-ploughing. Moreover, for the sinter containing unmodified MWCNTs, dark areas were observed on the worn surface in the BSE contrast, suggesting the presence of light elements such as carbon (CNTs) and oxygen. This was also confirmed by the chemical composition tests in the form of EDS maps. In the case of sinters with modified carbon nanotubes, in BSE contrast, the surface has an almost uniform gray color and the maps for the main components are also uniform. Slight differences in the distribution on the worn surface occur only for oxygen.

For both counter-specimens, the filling of the grooves formed during their preparation on 80-grit sandpaper was observed. The effect is to reduce the surface roughness of the counter-specimens. This indicates the self-healing ability of the friction surface by the sintered materials. In the case of the counter-specimen that works with the sinter containing unmodified nanotubes, a larger amount of sinter material was deposited on the Inconel^®^625 surface. On the basis of the observation of EDS maps, especially the distribution of copper on the surface of the counter-specimens, it can be concluded that, in the case of sinters containing modified nanotubes, a tribofilm is formed in the friction path, while in the case of sinters with unmodified MWCNTs, a thicker layer of copper is formed, which can be called a tribolayer. The overlap of copper and oxygen in the EDS maps suggests the formation of copper oxide.

**Figure 13:**
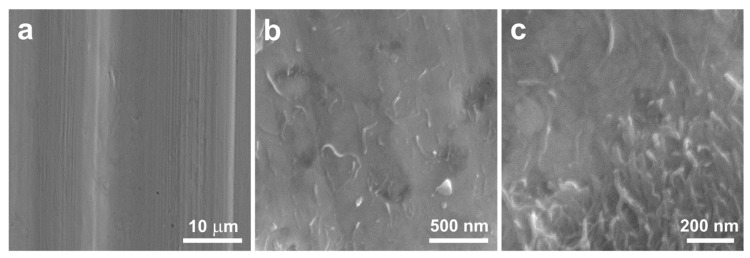
Worn surface of the 0.5 wt.% MWCNT/Cu sinters containing chemically modified nanotubes at different magnifications (**a**–**c**).

Figure 13 shows the worn surface of the sinters containing modified carbon nanotubes at a higher magnification. The worn surface is characterized by high smoothness with visible delicate microgrooves (Figure 13a). At high magnifications, carbon nanotubes, also in the form of single tubes, were observed on the worn surface of the sinters (Figure 13b,c). This proves the probable effect of the rolling motion of the MWCNTs across the mating friction pair. In addition, the presence of short tubes was observed. This proves their shortening during friction. Some nanotubes give the impression of coming out of the warp. It can be concluded that, in the case of these sinters, a controlled release of nanotubes was achieved during the friction test. Based on the tests of the worn surfaces of the friction pairs and analysis of [30,31,32,33,34], it was observed that the primary mechanism occurring between the friction pair involved the rolling of individual carbon nanotubes. The modified nanotubes released from the sinter during the test prevented direct metal-to-metal contact and reduced the adhesion between the contact surfaces of the friction pair.

In the case of sinters containing unmodified MWCNTs, their release from the metal matrix is easier and occurs in larger quantities at a given time. In these sinters, MWCNTs appear mainly in the form of agglomerates, which reduce the rolling effect and introduce a sliding effect. Because of the centrifugal force, MWCNT agglomerates are easier to remove from the friction path during the test. As a result, the metal-to-metal contact area increases and copper smears more easily on the surface of the counter-specimen, resulting in an increase in the value of the coefficient of friction. Moreover, due to the presence of copper on the surface of the counter-specimens (without carbon nanotubes because of their easier release), the friction conditions change, leading to intensified wear and an increased amount of worn debris.

Based on the tribological tests including the determination of the friction coefficients and observations of the surfaces of the friction pairs, it can be concluded that the modification of carbon nanotubes, combined with the method of preparing powder mixtures proposed in this work, allowed us to obtain sinters with more favorable tribological properties compared to previous studies [9,10], with a lower content of carbon nanotubes.

## 4. Conclusions

This paper describes powder metallurgy used to produce sinters with a copper matrix containing multi-walled carbon nanotubes modified by chemical oxidation. This research resulted in the formulation of the following conclusions:

− A positive effect was observed from the MWCNT modification and the proposed method of powder mixture preparation on the distribution of MWCNTs in the metal matrix and on the mechanical and tribological properties of the sinters. The sinters containing modified MWCNTs were characterized by a high repeatability of the test results.
− The addition of 0.5 wt.% MWCNTs to sintered copper increases its hardness by 19% compared to a pure copper sinter and is similar to sinters with unmodified carbon nanotubes, but the standard deviation for sinters containing modified MWCNTs is 5.3 times smaller compared to sinters containing unmodified MWCNTs.
− A comparison of the stress–strain characteristics of the examined micro-specimens indicates significantly improved mechanical properties in sinters containing modified MWCNTs, compared to both the sinters containing unmodified MWCNTs and the reference ones containing 100% pure copper.
− An over 40% increase in the average immediate tensile strength of the modified sinters was observed compared to the average strength of the 100% Cu reference ones and the sinters containing unmodified MWCNTs.
− The addition of modified 0.5 wt.% MWCNTs to sintered copper increases its yield strength by over 45% and 31%, compared to pure copper sinters and the sinters containing unmodified MWCNTs, respectively.
− Sinters containing modified MWCNTs are characterized by an approximately 15% higher Young’s modulus compared to sinters made of pure copper and sinters with unmodified MWCNTs.
− The modification of the nanotubes has allowed the production of a sinter that, during the analysis of the strain distributions in the uniaxial tensile test, behaves similarly to the 100% Cu reference one. Minimal areas of strain localization indicate a low probability of the surface fractures extending beyond the decohesion phase.
− For the sintered material containing modified MWCNTs, the coefficient of friction–time characteristics exhibits a high degree of stabilization and the average friction coefficient (0.27) is four times smaller than that of pure copper sinter.

## Figures and Tables

**Table 1:** Mechanical properties of the sinters.

Test	Compression			Tensile			Hardness	
Sinter	F_max_	Average E_mod_	W for F_max_	S_u_	S_y0_._2_	E	Average Hardness	Standard Deviation
	N	kN/mm	Nmm	MPa	MPa	MPa	HV5	
Cu (A)	8000	5.36 [7]	3948 [7]	87.8	50.7	65,432	26.35 [7]	-
0.5 wt.% unmodified MWCNTs/Cu (B)	8000	5.09	5095	89.2	56.7	65,237	31.31	5.85
0.5 wt.% modified MWCNTs/Cu (C)	8000	5.37	4787	125.9	74.3	75,665	31.30	1.10

## Data Availability

Data are contained within the article.
